# Acknowledgement to Reviewers of *Microarrays* in 2013

**DOI:** 10.3390/microarrays3010089

**Published:** 2014-02-28

**Authors:** Microarrays Editorial Office

**Affiliations:** MDPI AG, Klybeckstrasse 64, CH-4057 Basel, Switzerland

The editors of *Microarrays* would like to express their sincere gratitude to the following reviewers for assessing manuscripts in 2013:
Alzate, OscarGrigoriev, AndreySand, MichaelBaxter, Robert C.Grzebyk, DanielScharpf, Robert BBelin, Andrea CarmineGuerau-De-Arellano, MireiaSeroussi, E.Benn, PeterHess, Michael W.Settles, MattBicciato, SilvioJohnson, NickShanahan, Hugh P.Bort, Juan A. HernándezKarn, ThomasShaw-Smith, CharlesBovolenta, MatteoKeele, John W.Simplício, Ana LuísaBrown, Chad C.Khalyfa, AbdelnabySloan Bena, FrederiqueBruzzone, RobertoKotzot, DieterSöderberg, OlaBurroughs, A. MaxwellLabarge, MarkSokilde, RolfCarman, John G.Larson, Nicholas B.Spruck, CharlesChang, Jeffrey T.Leclerc, E.Srebniak, MalgorzataChechik, GalLin, TongTang, HaoChen, Chung-YungLiu, ZhenqiuTognini, PaolaChu, Jen-hwaLongy, MichelUnderhill, Gregory H.Coleman, William B.Lopez, ArturoVan Den Veyver, Ignatia B.Descombes, PatrickMascaux, CelineVonhof, MaartenDittmer, DirkMattheisen, ManuelWang, YingDubnau, JoshMerdes, GunterWapner, Ronald J.Eckel-Passow, Jeanette E.Meyer, Swanhild U.Weston, MichaelFardo, DavidMontazeri, ZahraWinegarden, NeilFredlund, ErikMüller, VolkmarYamada, MasumiGalluzzi, L.Nakas, ChristosYamamoto, NorioGeorge, SajiNovelli, AntonioYang, NingGiangreco, AdamNykter, MattiZavadil, JiriGirirajan, SanthoshOrr, MeganZhang, ChunxiangGlück, StefanPérez Jurado, Luis A.Zhang, YinanGouas, LaetitiaPergament, EugeneZink, DanieleGriffin, Darren K.Queiroz, Luzia Helena


